# Supplementary material for: Genetic risk for Alzheimer’s disease influences neuropathology via multiple biological pathways
Source: Brain Commun. 2020 Oct 12;2(2):fcaa167. doi: 10.1093/braincomms/fcaa167 (PMC7750986; doi:10.1093/braincomms/fcaa167)
Supplement: fcaa167_Supplementary_Data [file fcaa167_supplementary_data.zip › SupplementaryTables.docx]

Supplementary Table 1. No evidence of differential effect on neuropathology by Alzheimer's Disease PRS when stratified by *APOE* status

| **Neuropathology** **variable** | **Main effects** | | | | | | | | | **Interaction effects** | | | |
| --- | --- | --- | --- | --- | --- | --- | --- | --- | --- | --- | --- | --- | --- |
|  | ***APOE*** | | | | | | **Polygenic risk score** | | | **Number of E2 alleles x PRS** | | **Number of E4 alleles x PRS** | |
|  | **Number of E2 alleles** | | | **Number of E4 alleles** | | |  |  |  |  |  |  |  |
|  | **P-value** | **Coeff.** | **%VarExp** | **P-value** | **Coeff.** | **%VarExp** | **P-value** | **Coeff.** | **%VarExp** | **P-value** | **%VarExp** | **P-value** | **%VarExp** |
| **Braak stage tangle** | 0.0665 | -0.381 | 0.958 | 2.27E-23 | 1.13 | 15.1 | 3.78E-05 | 0.387 | 2.46 | 0.609 | 0.461 | 0.272 | 0.194 |
| **Thal amyloid stage** | 0.00146 | -0.61 | 1.54 | 6.49E-19 | 0.95 | 13.5 | 0.00269 | 0.257 | 0.589 | 0.0554 | 0.167 | 0.266 | 0.0559 |
| **CERAD stage** | 0.0172 | -0.342 | 1.99 | 4.01E-19 | 0.699 | 13.4 | 0.000459 | 0.223 | 2.19 | 0.626 | 0.425 | 0.359 | 0.0985 |
| **Braak Lewy body stage** | 0.941 | -0.021 | 0.0809 | 0.000147 | 0.58 | 2.59 | 0.0717 | 0.232 | 0.0964 | 0.338 | 0.198 | 0.0376 | 0.104 |
| **TDP-43** | 0.924 | -0.0312 | 0 | 0.00183 | 0.534 | 0 | 0.798 | 0.0376 | 0 | 0.712 | 0.0356 | 0.689 | 0.0418 |

Supplementary Table 2. *APOE* is associated with a shared effect on neurofibrillary tangles and β-amyloid. Results from regression models testing for associations between *APOE* or Alzheimer’s disease PRS and neuropathology while controlling for other measures of neuropathology.

|  |  | ***APOE*** | | | | | | **Polygenic risk score** | | |
| --- | --- | --- | --- | --- | --- | --- | --- | --- | --- | --- |
|  |  | **Number of E2 alleles** | | | **Number of E4 alleles** | | |  |  |  |
|  |  | **P-value** | **Coeff.** | **%VarExp** | **P-value** | **Coeff.** | **%VarExp** | **P-value** | **Coeff.** | **%VarExp** |
| **Covary for Braak NFT stage** | **Thal amyloid stage** | 0.00755 | -0.377 | 1.66 | 0.00202 | 0.257 | 13.7 | 0.394 | -0.0402 | 0.52 |
|  | **CERAD stage** | 0.0431 | -0.15 | 2.07 | 0.223 | 0.0515 | 13.4 | 0.855 | 0.00447 | 2.34 |
|  | **Braak Lewy body stage** | 0.863 | 0.0489 | 0.0874 | 0.0625 | 0.305 | 2.53 | 0.804 | 0.0236 | 0.093 |
|  | **TDP-43** | 0.952 | -0.0205 | 0.00101 | 0.238 | 0.221 | 0.389 | 0.84 | -0.0228 | 0.0114 |
| **Covary for Thal amyloid stage** | **Braak NFT stage** | 0.486 | 0.111 | 0.868 | 8.08E-07 | 0.459 | 16.5 | 0.000124 | 0.202 | 2.33 |
|  | **CERAD stage** | 0.951 | -0.00601 | 1.91 | 0.00265 | 0.169 | 13.7 | 0.00113 | 0.105 | 2.25 |
|  | **Braak Lewy body stage** | 0.609 | 0.146 | 0.0946 | 0.0999 | 0.276 | 2.57 | 0.396 | 0.081 | 0.18 |
|  | **TDP-43** | 0.73 | 0.12 | 0.0355 | 0.401 | 0.163 | 0.212 | 0.81 | 0.0275 | 0.0173 |
| **Covary for CERAD stage** | **Braak NFT stage** | 0.335 | 0.108 | 0.87 | 0.000159 | 0.239 | 15 | 0.0304 | 0.0799 | 2.54 |
|  | **Thal amyloid stage** | 0.0533 | -0.247 | 1.61 | 0.000336 | 0.265 | 13.6 | 0.379 | -0.0374 | 0.5 |
|  | **Braak Lewy body stage** | 0.695 | 0.11 | 0.0735 | 0.0896 | 0.273 | 2.51 | 0.745 | 0.0305 | 0.111 |
|  | **TDP-43** | 0.808 | 0.0832 | 0.0168 | 0.134 | 0.277 | 0.646 | 0.82 | -0.0257 | 0.0149 |
| **Covary for Braak Lewy body stage** | **Braak NFT stage** | 0.269 | -0.238 | 0.656 | 9.48E-19 | 1.07 | 15.5 | 3.10E-05 | 0.3 | 2.41 |
|  | **Thal amyloid stage** | 0.00506 | -0.541 | 1.44 | 5.37E-17 | 0.923 | 14.3 | 0.0327 | 0.138 | 0.576 |
|  | **CERAD stage** | 0.0442 | -0.29 | 1.82 | 6.32E-17 | 0.669 | 14 | 0.000664 | 0.163 | 2.01 |
|  | **TDP-43** | 0.806 | 0.0817 | 0.0176 | 0.00281 | 0.539 | 2.61 | 0.651 | 0.0509 | 0.0605 |
| **Covary for TDP-43** | **Braak NFT stage** | 0.16 | -0.294 | 0.787 | 7.90E-21 | 1.07 | 15.1 | 3.09E-05 | 0.285 | 2.16 |
|  | **Thal amyloid stage** | 0.0113 | -0.49 | 1.23 | 1.39E-17 | 0.919 | 13.2 | 0.0285 | 0.139 | 0.478 |
|  | **CERAD stage** | 0.0373 | -0.3 | 1.8 | 3.62E-17 | 0.659 | 13.7 | 0.000192 | 0.175 | 2.24 |
|  | **Braak Lewy body stage** | 0.988 | 0.00446 | 0.11 | 0.000263 | 0.573 | 3.02 | 0.443 | 0.0741 | 0.0544 |

Supplementary Table 3. *APOE* is not associated with cognition after controlling for neuropathology.

| **Analytical model** | **Cognitive/clinical variable** | **N** | ***APOE*** | | | | | |
| --- | --- | --- | --- | --- | --- | --- | --- | --- |
|  |  |  | **Number of E2 alleles** | | | **Number of E4 alleles** | | |
|  |  |  | **P-value** | **Coeff.** | **%VarExp** | **P-value** | **Coeff.** | **%VarExp** |
| **Test cognitive variables covarying for Braak tangle stage** | **CDR** | 549 | 0.724 | -0.045 | 0.36 | 0.196 | 0.093 | 9.96 |
|  | **MMSE** | 396 | 0.923 | -0.117 | 0.32 | 0.028 | -1.639 | 10.07 |
|  | **MOCA** | 228 | 0.258 | -2.006 | 0.06 | 0.735 | -0.406 | 3.11 |
| **Test Braak tangle stage covarying for cognitive variables** | **CDR** | 549 | 0.996 | 0.001 | 0.21 | 1.52E-09 | 0.640 | 14.08 |
|  | **MMSE** | 396 | 0.470 | -0.147 | 0.35 | 4.07E-05 | 0.511 | 12.47 |
|  | **MOCA** | 228 | 0.035 | -0.542 | 1.68 | 3.13E-06 | 0.797 | 12.27 |

Supplementary Table 4. Alzheimer’s disease PRS does not influence cognitive trajectory prior to death.

| **Time Variable** | **Clinical/ cognitive variable** | **Time** | | **AD PRS** | | **Time x AD PRS** | | **Number of individuals** | **Number of Observations** |
| --- | --- | --- | --- | --- | --- | --- | --- | --- | --- |
|  |  | **Coeff.** | **P-value** | **Coeff.** | **P-value** | **Coeff.** | **P-value** |  |  |
| **Time since study entry** | **CDR** | 3.50E-04 | 3.58E-24 | 0.0838 | 0.122 | 2.17E-05 | 0.467 | 564 | 1501 |
|  | **MMSE** | -2.69E-03 | 5.39E-22 | -0.662 | 0.162 | -4.59E-04 | 0.0426 | 406 | 975 |
|  | **MOCA** | -1.26E-03 | 0.0688 | -0.182 | 0.779 | 3.73E-04 | 0.557 | 235 | 419 |
| **Age** | **CDR** | 0.0106 | 0.0476 | 0.0681 | 0.871 | 6.41E-04 | 0.898 | 564 | 1501 |
|  | **MMSE** | -0.272 | 2.56E-08 | -5.17 | 0.177 | 0.0482 | 0.287 | 406 | 975 |
|  | **MOCA** | 0.0457 | 0.529 | -11.4 | 0.0635 | 0.134 | 0.0630 | 235 | 419 |

Supplementary Table 5. Braak neurofibrillary tangle stage alters cognitive trajectory prior to death.

| **Time variable** | **Clinical/ cognitive variable** | **Time** | | **Braak NFT stage** | | **Time x Braak NFT stage** | | **Number of individuals** | **Number of Observations** |
| --- | --- | --- | --- | --- | --- | --- | --- | --- | --- |
|  |  | **Coeff.** | **P-value** | **Coeff.** | **P-value** | **Coeff.** | **P-value** |  |  |
| **Time since study entry** | **CDR** | 6.87E-05 | 0.327 | 0.355 | 7.30E-42 | 6.77E-05 | 3.99E-05 | 550 | 1461 |
|  | **MMSE** | 6.34E-04 | 0.181 | -2.58 | 7.27E-26 | -1.05E-03 | 3.90E-15 | 397 | 957 |
|  | **MOCA** | -4.50E-04 | 0.733 | -2.90 | 1.50E-14 | -3.87E-04 | 0.323 | 228 | 412 |
| **Age** | **CDR** | -0.0375 | 7.74E-06 | -0.571 | 6.69E-04 | 0.0120 | 1.23E-08 | 550 | 1461 |
|  | **MMSE** | 0.0916 | 0.253 | 4.68 | 0.0107 | -0.0939 | 3.63E-05 | 397 | 957 |
|  | **MOCA** | 0.159 | 0.152 | 0.489 | 0.871 | -0.0423 | 0.253 | 228 | 412 |

Supplementary Table 6. *APOE* E4 is no longer associated influences cognitive decline prior to death after controlling for Braak neurofibrillary tangle stage

| **Time variable** | **Clinical/ cognitive variable** | **Time** | | ***APOE*** | | | | **Interaction (Time x *APOE*)** | | | | **Number of individuals** | **Number of observations** |
| --- | --- | --- | --- | --- | --- | --- | --- | --- | --- | --- | --- | --- | --- |
|  |  |  |  | **Number of E2 alleles** | | **Number of E4 alleles** | | **Time x E2** | | **Time x E4** | |  |  |
|  |  | **Coeff.** | **P-value** | **Coeff.** | **P-value** | **Coeff.** | **P-value** | **Coeff.** | **P-value** | **Coeff.** | **P-value** |  |  |
| **Time since study entry** | **CDR** | 8.98E-05 | 0.229 | 0.111 | 0.423 | 0.107 | 0.173 | -9.27E-05 | 0.280 | 3.45E-05 | 0.531 | 549 | 1459 |
|  | **MMSE** | 1.05E-03 | 0.037 | 0.666 | 0.567 | -0.635 | 0.377 | -8.90E-04 | 0.118 | -1.11E-03 | 0.005 | 396 | 956 |
|  | **MOCA** | -2.41E-03 | 0.121 | -2.086 | 0.190 | 0.807 | 0.460 | 2.33E-03 | 0.089 | -3.60E-03 | 0.020 | 228 | 412 |
