# Supplementary material for: Genetic risk for Alzheimer’s disease influences neuropathology via multiple biological pathways
Source: Brain Commun. 2020 Oct 12;2(2):fcaa167. doi: 10.1093/braincomms/fcaa167 (PMC7750986; doi:10.1093/braincomms/fcaa167)
Supplement: fcaa167_Supplementary_Data [file fcaa167_supplementary_data.zip › SupplementaryFigures.docx]

**Supplementary Figures**

Supplementary Fig. 1. Scatterplot of first two genetic principal components of BDR samples merged with HapMap. Each point represents either a BDR or HapMap sample, where HapMap samples are coloured by their known ethnicity. HapMap samples were used to identify BDR samples that were not of European ancestry. The solid lines define the threshold used to select BDR samples for analysis. BDR – Brains for Dementia Research


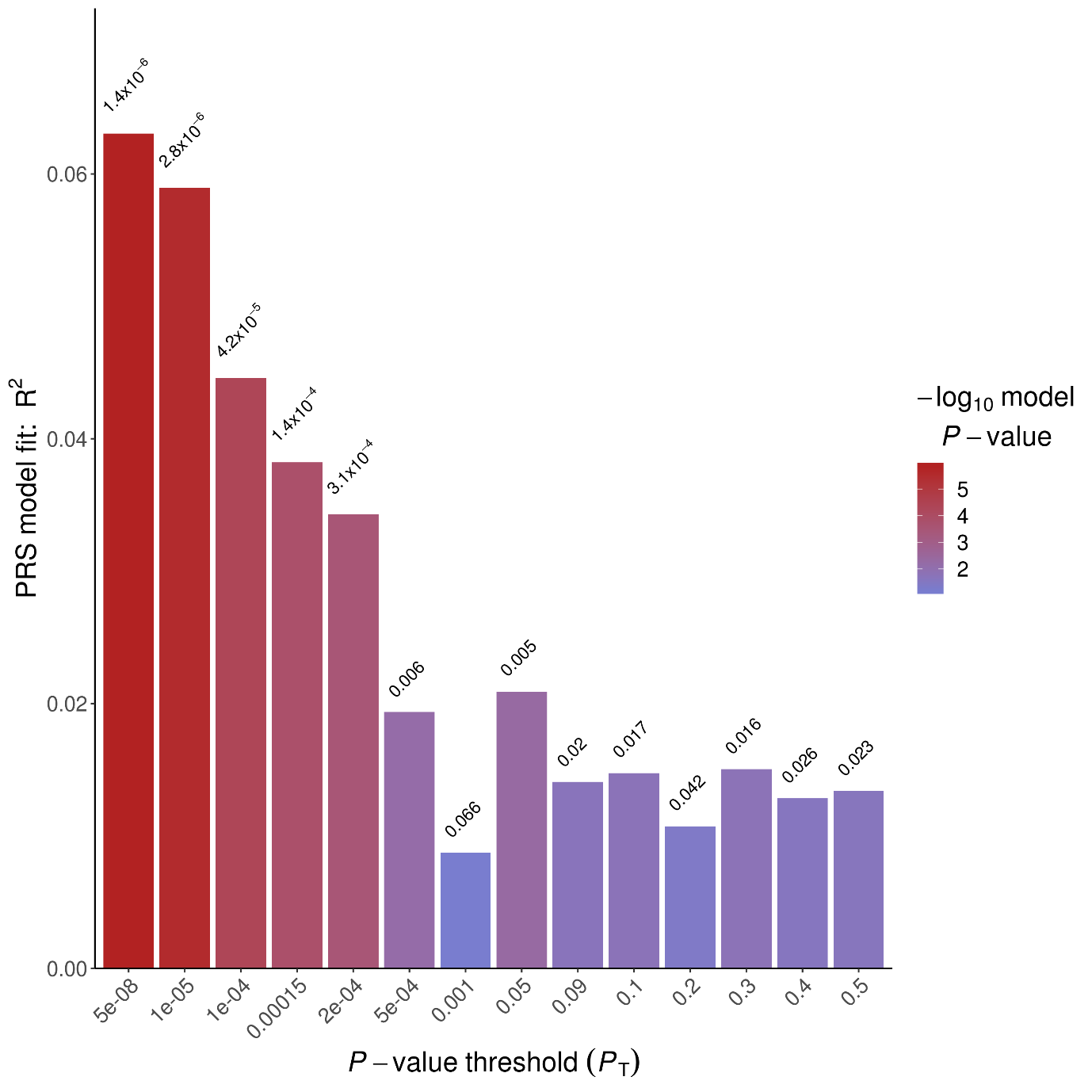


Supplementary Fig. 2. Bar plot of Alzheimer’s disease PRS tested against Alzheimer’s disease case control status in BDR cohort. Shown are multiple PRS calculated at different P-value thresholds (P_T_) tested against Alzheimer’s disease case control status. The y-axis indicates the proportion of variance explained and the colour of the bar indicates the P-value of the association, which is also provided above each bar. PRS – polygenic risk score; BDR – Brains for Dementia Research Cohort.

Supplementary Fig. 3. Heatmap of correlations between semi-quantitative measures of Neuropathology. NFT- neurofibrillary tangles, LB – lewy body.
